# DNA barcoding and surveillance sampling strategies for *Culicoides* biting midges (Diptera: Ceratopogonidae) in southern India

**DOI:** 10.1186/s13071-016-1722-z

**Published:** 2016-08-22

**Authors:** Lara E. Harrup, Swathi Laban, Bethan V. Purse, Yarabolu Krishnamohan Reddy, Yella Narasimha Reddy, Sonnahallipura Munivenkatappa Byregowda, Naveen Kumar, Kondappa Muniramaiah Purushotham, Shrikant Kowalli, Minakshi Prasad, Gaya Prasad, Alison A. Bettis, Rien De Keyser, James Logan, Claire Garros, David Gopurenko, Glenn Bellis, Karien Labuschagne, Bruno Mathieu, Simon Carpenter

**Affiliations:** 1 Vector-borne Viral Diseases Programme, The Pirbright Institute, Ash Road, Woking, Surrey GU24 0NF UK; 2 Vaccine Research Centre-Viral Vaccines, Centre for Animal Health Studies, Tamil Nadu Veterinary and Animal Sciences University, Madhavaram Milk Colony, Chennai, 600 051 India; 3 Centre for Ecology and Hydrology, Benson Lane, Crowmarsh Gifford, Wallingford, Oxfordshire OX10 8BB UK; 4 Department of Veterinary Microbiology, College of Veterinary Science, Rajendranagar, Hyderabad, 500030 Andhra Pradesh India; 5 Institute of Animal Health and Veterinary Biologicals, Hebbal, 560024 Bengaluru India; 6 Department of Animal Biotechnology, Lala Lajpat Rai University of Veterinary and Animal Sciences, College of Veterinary Science, Hisar, 125004 Haryana India; 7 Indian Council Agricultural Research, New Delhi, 110 001 India; 8 Department of Disease Control, London School of Hygiene and Tropical Medicine, London, WC1E 7HT UK; 9 Cirad, UMR15 CMAEE, F-34398 Montpellier, France; 10 INRA, UMR1309 CMAEE, F-34398 Montpellier, France; 11 NSW Department of Primary Industries, PMB, Wagga Wagga Agricultural Institute, Wagga Wagga, NSW 2650 Australia; 12 Graham Centre for Agricultural Innovation, Locked Bag 588, Wagga Wagga, NSW 2678 Australia; 13 Department of Agriculture, Fisheries and Forestry, Winnellie, Australia; 14 Onderstepoort Veterinary Institute, Agricultural Research Council-Onderstepoort Veterinary Institute, PVVD, ZA-0110 Onderstepoort, South Africa; 15 Department of Zoology and Entomology, University of Pretoria, ZA-0002 Pretoria, South Africa; 16 Institut de Parasitologie et de Pathologie tropicale de Strasbourg (IPPTS), EA7292, Faculté de Médecine, 3 rue Koeberlé, F-67000 Strasbourg, France

**Keywords:** *Culicoides*, Bluetongue virus, Arbovirus, DNA barcode, BOLD, COI, LED, Surveillance

## Abstract

**Background:**

*Culicoides* spp. biting midges transmit bluetongue virus (BTV), the aetiological agent of bluetongue (BT), an economically important disease of ruminants. In southern India, hyperendemic outbreaks of BT exert high cost to subsistence farmers in the region, impacting on sheep production. Effective *Culicoides* spp. monitoring methods coupled with accurate species identification can accelerate responses for minimising BT outbreaks. Here, we assessed the utility of sampling methods and DNA barcoding for detection and identification of *Culicoides* spp. in southern India, in order to provide an informed basis for future monitoring of their populations in the region.

**Methods:**

*Culicoides* spp. collected from Tamil Nadu and Karnataka were used to construct a framework for future morphological identification in surveillance, based on sequence comparison of the DNA barcode region of the mitochondrial cytochrome *c* oxidase I (*COI*) gene and achieving quality standards defined by the Barcode of Life initiative. Pairwise catches of *Culicoides* spp. were compared in diversity and abundance between green (570 nm) and ultraviolet (UV) (390 nm) light emitting diode (LED) suction traps at a single site in Chennai, Tamil Nadu over 20 nights of sampling in November 2013.

**Results:**

DNA barcode sequences of *Culicoides* spp. were mostly congruent both with existing DNA barcode data from other countries and with morphological identification of major vector species. However, sequence differences symptomatic of cryptic species diversity were present in some groups which require further investigation. While the diversity of species collected by the UV LED Center for Disease Control (CDC) trap did not significantly vary from that collected by the green LED CDC trap, the UV CDC significantly outperformed the green LED CDC trap with regard to the number of *Culicoides* individuals collected.

**Conclusions:**

Morphological identification of the majority of potential vector species of *Culicoides* spp. samples within southern India appears relatively robust; however, potential cryptic species diversity was present in some groups requiring further investigation. The UV LED CDC trap is recommended for surveillance of *Culicoides* in southern India.

**Electronic supplementary material:**

The online version of this article (doi:10.1186/s13071-016-1722-z) contains supplementary material, which is available to authorized users.

## Background

Bluetongue (BT) is an economically important disease of sheep in the southern Indian states of Tamil Nadu, Karnataka, Telangana and Andhra [1]. Outbreaks of BT have a major impact on sheep rearing in southern India due to the high proportion of subsistence level sheep farmers in the region, who have limited access to vaccines and palliative care for their livestock [2]. The aetiological agent of BT, bluetongue virus (BTV), is biologically transmitted between ruminant hosts by competent vectors of the genus *Culicoides* (Diptera: Ceratopogonidae) [3]. In India, the epidemiology of BTV is highly complex, potentially involving multiple vector species and with at least 21 BTV serotypes identified by serology [1], some of which may have been introduced during efforts to improve ruminant production [4].

Seven putative BTV vector species are known to occur in India (*Culicoides actoni* Smith, 1929; *C. brevitarsis* Kieffer, 1917; *C. dumdumi* Sen & Das Gupta, 1959; *C. fulvus* Sen & Das Gupta, 1959; *C. imicola* Kieffer, 1913; *C. oxystoma* Kieffer 1910 and *C. peregrinus* Kieffer, 1910) [1, 5–7], although this implication is derived primarily from vector competence data collected in other countries. *Culicoides imicola* and *C. oxystoma* have been reported to extend across the Afrotropical, Saharo-Arabian and Oriental regions [8, 9] (geographic regions defined as per Holt et al. [10]). In contrast, *C. actoni*, *C. brevitarsis*, *C. dumdumi, C. fulvus* and *C. peregrinus* have been recorded in the Australian, Oceanian and Oriental region [11–15], but not in the Saharo-Arabian and Afrotropical regions. The combination of multiple potential vector species and a huge diversity of BTV strains [16, 17] makes India one of the most challenging areas in which to dissect transmission cycles and highlights the importance of this region due to it sharing features of the Afrotropical, Saharo-Arabian, Oriental and Australasian ecozones [6]. While broad relationships between *Culicoides* spp. abundance and transmission have been suggested [1], these remain very poorly defined and hence unpredictable.

The *Culicoides* fauna of the Oriental region has been the focus of an authoritative taxonomic review based on morphology [18]. Wirth & Hubert’s review [18], however, did not extend to a comprehensive review of the *Culicoides* fauna of the Indian subcontinent and the *Culicoides* fauna of India has only been subject to sporadic morphological studies, e.g. Das Gupta [19, 20]. Checklists of Indian species of *Culicoides* have been produced [21–23]; however, many contain misidentifications and synonymous species [22, 23] and/or propose new species with no supporting taxonomic data [23], rendering them of limited use with regard to compiling biodiversity inventories or investigating *Culicoides*-borne arbovirus epidemiology. In addition, molecular DNA analyses of the Indian *Culicoides* fauna are limited to a single DNA barcode [24] report focussed on five species sampled from a single location, with little comment regarding the specificity of the DNA barcodes relative to other *Culicoides* species or populations [25]. Further DNA barcode and molecular studies are required to underpin morphological studies of the *Culicoides* fauna of India, as has been accomplished elsewhere to clarify species-level taxonomic descriptions [26, 27].

Creating a fundamental base for *Culicoides* species diagnostics in India is a prerequisite for dissecting BTV epidemiology accurately in this country. Wider questions also exist regarding the phylogenetic and taxonomic relationships of *Culicoides* populations in southern India with those from other regions including the degree of haplotype connectivity between global populations of vector species. Attempts to resolve these questions may be achieved through the development of morphological and genetic datasets of *Culicoides* spp. from India that are comparable with those being produced elsewhere (for review, see Harrup et al. [28]).

In addition to accurate species identification, a second fundamental requirement for accurate surveillance of *Culicoides* populations in southern India is the selection of appropriate monitoring tools. Systematic sampling of *Culicoides* populations using light-suction trapping next to livestock has been used to demarcate geographic and temporal risk of BTV transmission in Europe, Africa and Australia [29–33]. In both Europe and Africa, *Culicoides* spp. surveillance programmes are reliant upon the use of ultraviolet (UV) (~390 nm peak wavelength) light-suction traps, which have previously been shown to be highly effective at collecting *C. imicola* [33], the principle vector of BTV in Africa and the Mediterranean Basin. In Australia, however, green light emitting diode (LED) (~520 nm peak wavelength) light-suction traps are used to collect *Culicoides* spp. as part of the National Arbovirus Monitoring Program [34]. The preference for using green light for the collection of *Culicoides* spp. in Australia is the result of field studies demonstrating that the primary BTV vector species in this region, *C. brevitarsis*, has a greater sensitivity to this wavelength than to other colours including UV [31]. As both *C. imicola* and *C. brevitarsis* occur in India [18], it is important to define prior to the initiation of wide-scale surveillance project which light wavelength is most effective for surveillance of these and other *Culicoides* spp. in southern India. In addition, the logistical challenge of establishing field surveys and specifically the limited access to reliable mains electricity for recharging of batteries used to power light-suction trap surveys has limited adult *Culicoides* surveillance in India to single state studies involving one or a few sites. These challenges must be assessed prior to the deployment of large-scale surveillance programmes in order to confirm the selected equipment is both effective at the collection of *Culicoides* spp. and robust enough to withstand local conditions.

In this study we DNA barcoded *Culicoides* spp. collected across southern India and assessed the phylogenetic utility of these DNA barcodes to provide species identifications in agreement with morphology-based taxonomic identifications. We included publically available DNA barcodes from global replicates of targeted species in our analyses to determine if the populations in India contained unique and/or cosmopolitan genetic diversity. In addition, we also assess the use of two commercially available LED Center for Disease Control (CDC) light-suction traps [35, 36] as a precursor to wide-scale surveillance of *Culicoides* spp. in southern India. In the absence of logistically feasible trapping methods that are reflective of biting rates on ruminants, the key considerations for such a surveillance scheme are that the selected light-suction traps collect a wide-diversity of *Culicoides* spp. and at a sufficient abundance to consistently discern seasonal patterns in abundance.

## Methods

### Specimen selection and morphological identification

**Fig. 1:**
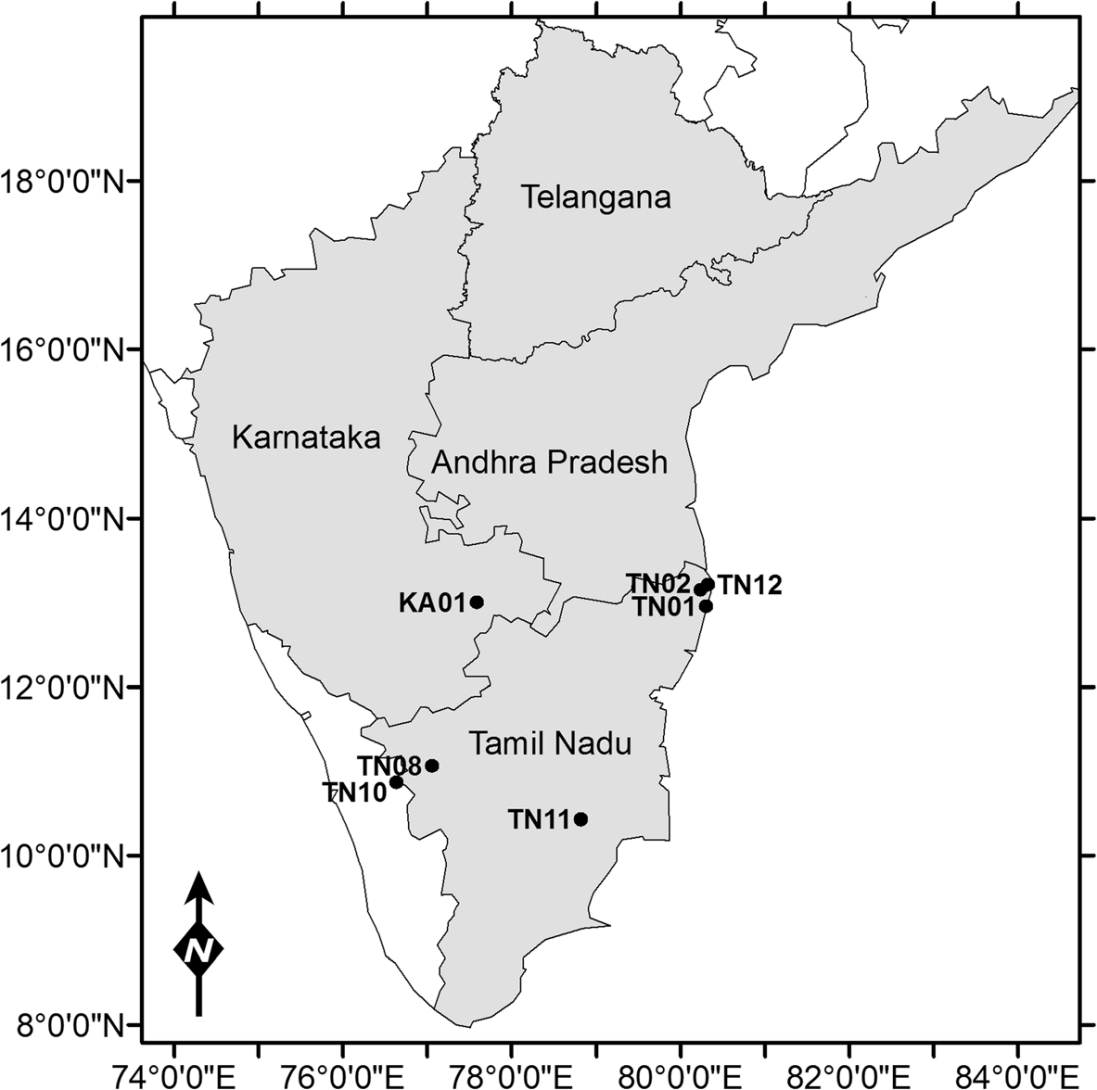
Geographical location of sites from which *Culicoides* spp. specimens selected for genetic analysis were collected: TN01 (*n* = 16); TN02 (*n* = 16); TN08 (*n* = 10); TN10 (*n* = 14); TN11 (*n* = 9); TN12 (*n* = 3); KA01 (*n* = 5)

**Fig. 2:**
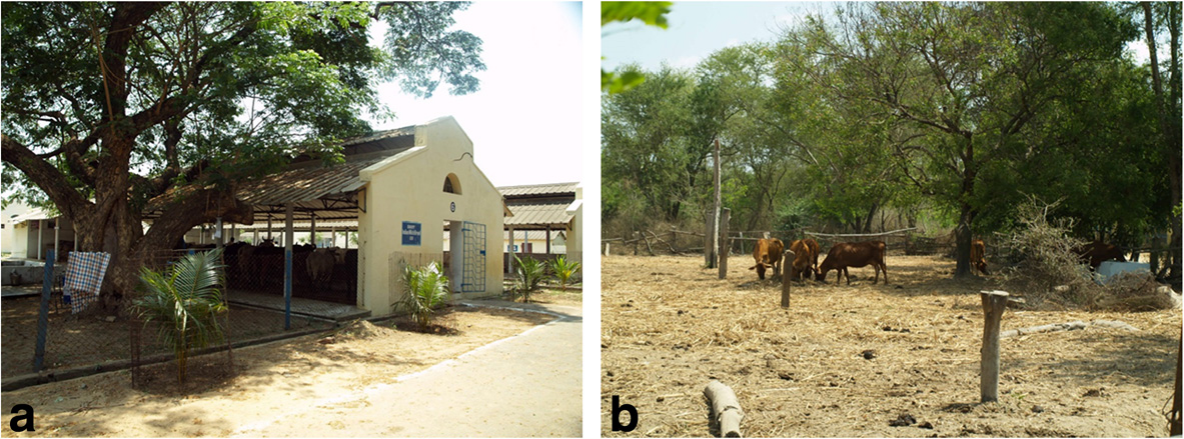
Collection sites, examples of typical semi-intensive farming habitat found in southern India. **a** TN01 (Chennai, Tamil Nadu, India); **b** TN02 (Kattupakkam, Tamil Nadu India)

**Fig. 3:**
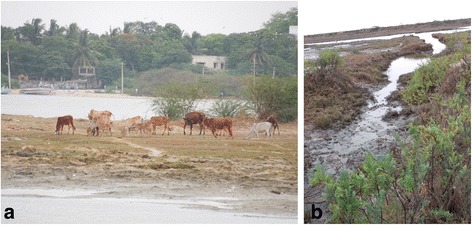
Collection Site TN12 (Chennai, Tamil Nadu) example of **a** coastal inter-tidal habitat with **b** cattle grazing, an example of typical subsistence farming habitat found in coastal regions of southern India

Seventy-three *Culicoides* specimens from seven sites and representing 12 morphologically identified species (Additional file 1: Table S1) and one currently unknown species were selected for genetic characterisation (Fig. 1) [TN01 (*n* = 16); TN02 (*n* = 16); TN08 (*n* = 10); TN10 (*n* = 14); TN11 (*n* = 9); TN12 (*n* = 3); KA01 (*n* = 5)]. Sites TN01 and TN12 were located near Chennai, Tamil Nadu, TN02 near Kattupakkam, Tamil Nadu and TN11 near Erode, Tamil Nadu (Fig. 1), and are all located in areas characterised as having tropical wet and dry climates [37], being particularly effected by the north east monsoon. Site TN08 is located near Pudukottai, Tamil Nadu, a semi-arid area with high temperatures throughout the year and relatively low rainfall. Site TN10 is near Ooty in Tamil Nadu, an area with a subtropical highland climate [37]. Site KA01 is located near Bangalore, Karnataka in an area with a tropical savannah climate [37] where the southwest monsoon has a greater influence than the northeast monsoon on climate conditions. Sites TN01, TN02, TN08, TN10, TN11 and KA01 were all located in areas principally utilised for subsistence and/or semi-intensive sheep, goat, cattle and buffalo rearing (Fig. 2). Site TN12 is located in an area of coastal inter-tidal marsh with low-level subsistence sheep, goat, cattle and buffalo farming present in the area (Fig. 3).

Specimens selected for genetic analysis were collected either using an UV LED CDC light-suction trap (Model 2770, 390 nm peak wavelength: BioQuip Products, Rancho Dominguez, CA, USA) or a sweep net and stored in 70 % ethanol prior to identification. *Culicoides* specimens were selected for genetic characterisation following preliminary identification using an SMZ-140 stereomicroscope (Motic, Barcelona, Spain) and the keys of Boorman [38], Gangopadhyay & Das Gupta [22] and Wirth & Hubert [18], and the descriptions of Majumdar et al. [39] and Nandi & Mazumdah [40, 41].

### Molecular identification

#### DNA extraction

Total DNA was extracted from individual *Culicoides* specimens using a non-destructive technique [42]. Specimens were individually incubated in 200 μl of DXT Tissue Digest Reagent (QIAGEN, Crawley, UK) with 1 % Proteinase K (QIAGEN) for 16 h at 40 °C. *Culicoides* were then stored at 4 °C in 70 % ethanol prior to slide mounting. The remaining tissue digest solution was incubated at 70 °C for 15 min to inactivate the proteinase K, and then ethanol-precipitated to remove PCR inhibitors using Pellet Paint® Co-Precipitant (Merck Millipore, Darmstadt, Germany) to improve DNA yield. The purified DNA extractions were re-suspended in 100 μl of 10 mM Tris HCl, pH 8.0 (Buffer EB: QIAGEN) and stored at 4 °C.

#### COI DNA barcode assay

Amplification of a 658 bp fragment of the mitochondrial *COI* gene barcoding region [24] was achieved by polymerase chain reaction (PCR) using an Eppendorf® Mastercycler® Pro (Eppendorf®, Chennai, India) thermal cycler. Reactions were performed in a total volume of 25 μl consisting of 2.5 μl nuclease-free water (QIAGEN), 12.5 μl QIAGEN TopTaq Master Mix, 2.5 μl CoralLoad Concentrate (QIAGEN), 1.25 μl of the 20 μM forward primer LCO1490 (5′-GGT CAA CAA ATC ATA AAG ATA TTG G-3′ [43]), 1.25 μl of the 20 μM reverse primer HCO2198 (5′-TAA ACT TCA GGG TGA CCA AAA AAT CA-3′ [43]) and 5.0 μl of template DNA (approximately 5–25 ng DNA) for each reaction. Positive and negative controls for the amplification reactions were carried out at every PCR round. The PCR cycling conditions were as follows: an initial denaturation step at 94 °C for 3 min followed by 35 cycles of 94 °C for 30 s, 46 °C for 30 s, 72 °C for 1 min, and a final extension step at 72 °C for 10 min. Reactions were stored at 4 °C until further processing. PCR products were visualised through electrophoresis on 2 % (*w/v*) pre-cast agarose gels containing ethidium bromide (E-Gel™ 48 gels: ThermoFisher Scientific, UK) run for 8 min. Successful amplification of the *COI* DNA barcode region was indicated by the presence of a band at approximately 720 bp, identified by comparison with E-Gel® Low Range Quantitative DNA Ladder (100–2000 bp: ThermoFisher Scientific).

#### PCR purification and COI sequencing

Dimer formation from the primers was not observed and purification of the remaining PCR product was performed using the MinElute® PCR purification kit (QIAGEN) following manufacturers recommended guidelines (v. 03/2008). The resulting products were sent for bi-directional sequencing using primers HCO2198 (reverse) and LCO1490 (forward) at a commercial facility (Eurofins, Bangalore, India). The resulting electropherograms were edited and forward and reverse sequences assembled and trimmed to remove primer sequence using CodonCode Aligner v. 5.1.5 (CodonCode Aligner, Centerville, MA, USA). Corresponding specimen collection data and DNA sequences including electropherograms have been made publically available via the Barcode of Life Data System (BOLD) [44] as dataset DS-CULIN (10.5883/DS-CULIN) and DNA sequences are also available in the GenBank database under accession numbers KT307786–KT307856.

### Phylogenetic analysis

Consensus sequences were compared to previously published sequences in the GenBank database using the standard nucleotide BLAST tool [45], in addition to comparison to as yet unreleased sequence data in the BOLD database [44] using the Barcode Identification Engine in BOLD v3. Sequences from GenBank included in the phylogenetic analysis (*n* = 196) are listed in Additional file 1: Table S1. GenBank sequences were included in the analysis to assess if the morphological identifications made within this study were conspecific with those made from other geographical regions and were not confounded with species morphologically similar enough to result in misidentification, e.g. *C. brevitarsis* and *C. bolitinos* Meiswinkel, 1989 [26]. All sequences were aligned using MUSCLE [46] and quality checked using GUIDANCE [47] (100 bootstraps). All included sequences were aligned with a high degree of confidence (GUIDANCE alignment score > 0.999). The general time reversible model with gamma-distribution rates plus invariant Sites (GTR + Γ + I) was identified using ModelTest2 [48, 49] v 2.1.4 as the optimal model of nucleotide substitution in the alignment (outgroup excluded), based on the lowest Bayesian Information Criterion (BIC) and Akaike Information Criterion (AIC) scores.

The phylogenetic relationships among taxa were resolved using a Bayesian Inference (BI) approach [50, 51], with the topology rooted on the partial COI sequence of *Anopheles gambiae* Giles, 1902 (NC002084 [52]). The BI tree was constructed using MrBayes v.3.2.2 [50, 51] and twenty million tree generations in four chains were run, sampling every 1000th and discarding the first 25 %, before constructing a 50 % majority rule consensus tree reporting Bayesian posterior probabilities. The absence of indicators of a lack of convergence in the final consensus BI topology was confirmed via the examination of the sampled Markov chain Monte Carlo tree topologies using AWTY [53].

Relationships between the observed haplotypes within the *C. brevitarsis - C. asiana* Bellis, 2014 (*nomen novum* for *C. asiatica* Bellis [54] preoccupied by *C. asiaticus* Gutsevich & Smatov 1966; specimens redescribed by Bellis et al. [26]), *C. imicola* and *C. oxystoma* clades were assessed by constructing Median-Joining networks. Roehl haplotype data files (RDF) were created with DnaSP v.5.10 [55] and imported into Network v.4.6.1.2 [56] and networks were calculated with the Median-Joining algorithm [57] with equal weights for all characters, using maximum parsimony [58] post-processing. Uncorrected intra- and inter-specific percentage sequence distances were generated using the packages Spider v 1.3-0 [59] and Ape v.3.2 [60], implemented in R v.3.1.2 [61]. Missing nucleotides were treated in all sequence comparisons using a pairwise deletion option.

### Morphological voucher specimens

Following DNA extraction specimens were then individually dissected and slide mounted in Euparal following the techniques of Nevill & Dyce [62]. Mounted specimens were re-examined following mounting and identifications confirmed using the keys of Boorman [38], Gangopadhyay & Das Gupta [22] and Wirth & Hubert [18], and the descriptions of Majumdar et al. [39] and Nandi & Mazumdah [40, 41].

### Light-suction trap comparison

Commercially produced, modified CDC design light-suction traps fitted with LEDs were compared (Model 2770: BioQuip Products, Rancho Dominguez, CA, USA). The LED platforms emitted peak light wavelengths of either 390 nm (UV) or 570 nm (Green) and were powered by 6 V batteries. An inline photo-switch (BioQuip) was also used on each trap to standardise collection periods, reduce battery consumption during the trial and replicate their planned use in the surveillance programme. Two locations over 50 m apart at site TN01 (Fig. 1) were chosen for this study (trap height approximately 1.5 m), both of which were in close proximity to ruminant livestock (1*–*5 m from cattle; 40*–*50 m from sheep). Trapping was conducted during November 2013, during the predicted peak BTV transmission period, for 20 nights, with the positions of the green and UV LED CDC traps rotated between the two sites on alternate nights. Overnight collections (approximately three hours before sunset to three hours after sunrise) were made into water containing a drop of detergent and then transferred the following morning to 70 % ethanol for storage prior to identification.

Following collection, specimens of *Culicoides* were identified morphologically under a stereomicroscope using keys [18, 63] and comparison to reference specimens from the local area produced as part of the phylogenetic analysis conducted within this study. Specimens were sexed and females were further separated based on their abdominal pigmentation status (unpigmented/ nulliparous, blood-fed, gravid, or pigmented/ parous) [64, 65]. In large collections (estimated by the investigator to contain more than 1,000 *Culicoides* spp. specimens), a standardised process of subsampling was used to estimate the abundance and diversity of *Culicoides* spp. present [66]. In summary, for samples which were subsampled, insect collections were washed with water through a series of stainless steel test sieves (3.35 mm, 2.00 mm, 1.00 mm and 300 μm mesh diameter). The contents of the 300 μm sieve were then transferred to a weigh boat and weighed. Successive 1 g portions of the sample in the weigh boat were then sorted and identified. Successive 1 g portions were taken and completely sorted and identified until at least 650 individuals of *Culicoides* (any species) had been identified. The total number of specimens of a particular species of *Culicoides* in the original sample was then estimated as equal to the [(Total weight of the sieved sample/ number of grams of sample identified) × number of *Culicoides* of the species of interest identified in the subsample].

### Statistical analysis

Relationships between female *Culicoides* abundance and five fixed effects were examined using generalised linear mixed models with a negative binomial error distribution (fitted using the glmmadmb package v 0.7.5 [67, 68] in R [61]), three main effects of *light type*, *position* and *species* (including the five most abundant species) and two possible interactions (*species* × *light type*, to reflect that light types may be more attractive to some species than others and *species* × *position*, to reflect that a trap position may have been closer or further from a larval development site which may vary by *Culicoides* species). In all models, a random effect of *trapping day* was used. All possible combinations of the fixed effects were examined (including an intercept only model) and the model with the lowest AIC [69] was selected. The same procedure was followed for males, with the exception that a zero-inflated negative binomial error distribution was used to account for the higher proportion of zero catches for males which resulted in significantly overdispersed residuals in ordinary negative binomial models. The diversity of *Culicoides* species collected by the two trap types, i.e. the species richness, was further compared using the Margalef’s index, such that Margalef’s index = (*S* - 1)/*ln N*, where *S* is the total number of species collected in a sample, i.e. one trap collection, *N* is the total number of individuals in the sample and *ln* is the natural logarithm [70].

## Results

### Phylogenetic analysis

Full length primer truncated DNA barcode sequences of 658 bp were recovered from 71 of the 73 specimens sampled from India, representing 12 morphologically identified species: *C. actoni*; *C. anophelis* Edwards, 1922; *C. brevitarsis*; *C. huffi* Causey, 1938; *C. imicola*; *C. innoxius* Sen & Das Gupta, 1959; *C. kepongensi*s Lee, 1988; *C. mesghalii* Navai, 1973; *C. oxystoma*; *C. peliliouensis* Tokunaga, 1936; *C. peregrinus*; and *C. similis* Carter, Ingram & Macfie, 1920 (Additional file 1: Table S2), and one currently unidentified species. Amino acid frame shifts and stop codons were not evident among sequence translations, indicating pseudogenes were not likely to be included in the alignments. The *COI* sequences obtained from the GenBank (Additional file 1: Table S1) overlapped the alignment of the sequences generated in this study by between 434 and 658 bp.

**Fig. 4:**
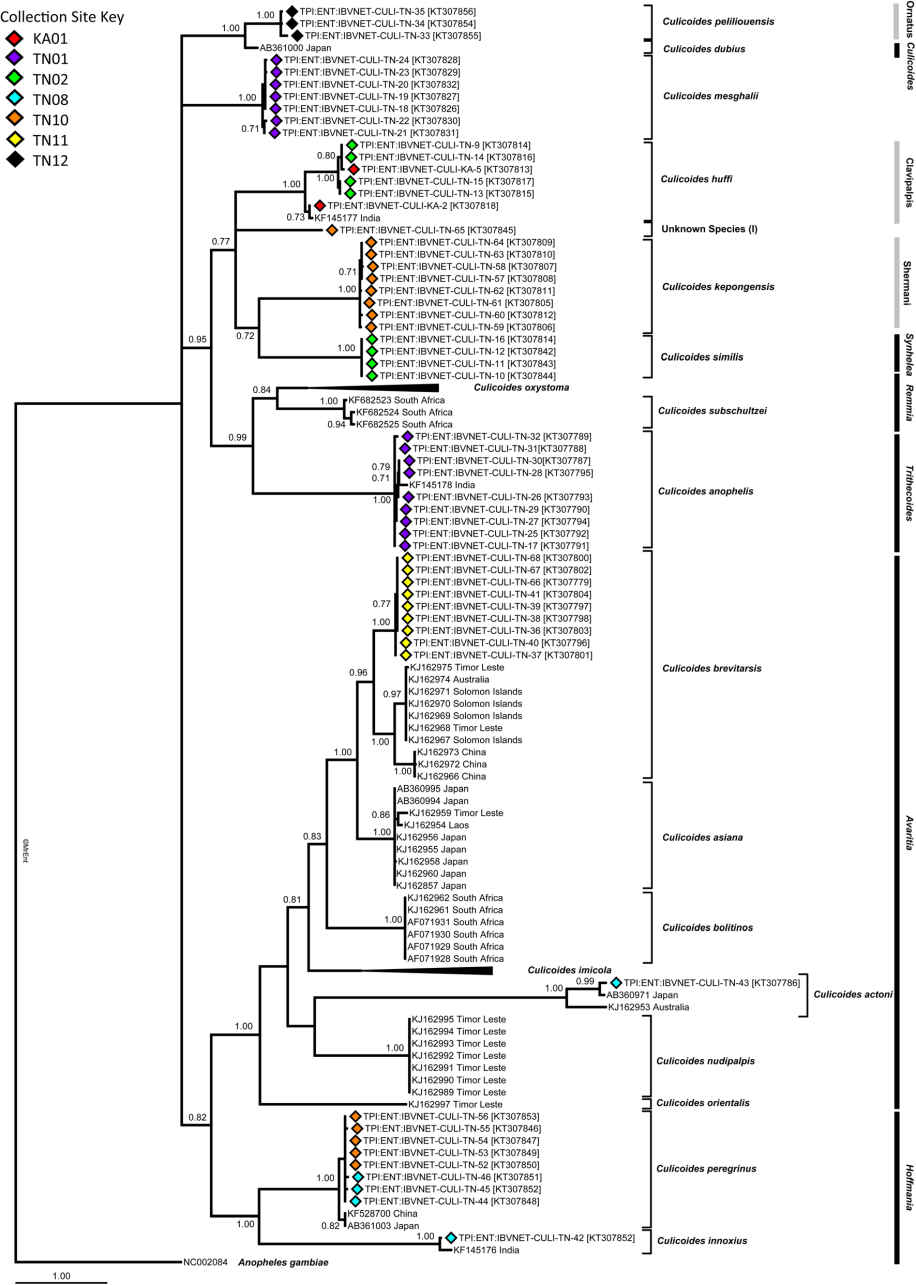
Bayesian Inference phylogenetic tree inferred from *COI* DNA barcode sequences with species, and subgeneric (*thick black line*, *italic font*) or species group (*thick black line*, *Roman font*) indicated. Bayesian posterior probability node support values greater than 0.7 shown. Coloured diamonds indicate specimens from the IBVNet project coloured by collection site (KA1: *red*; TN01: *purple*; TN02: *green*; TN08: *blue*; TN10: *orange*; TN11: *yellow*; TN12: *black*) with specimen number followed by GenBank accession number in brackets. *Culicoides imicola* and *C. oxystoma* clade summarised, see Figs. 5 and 6 for further details

**Fig. 5:**
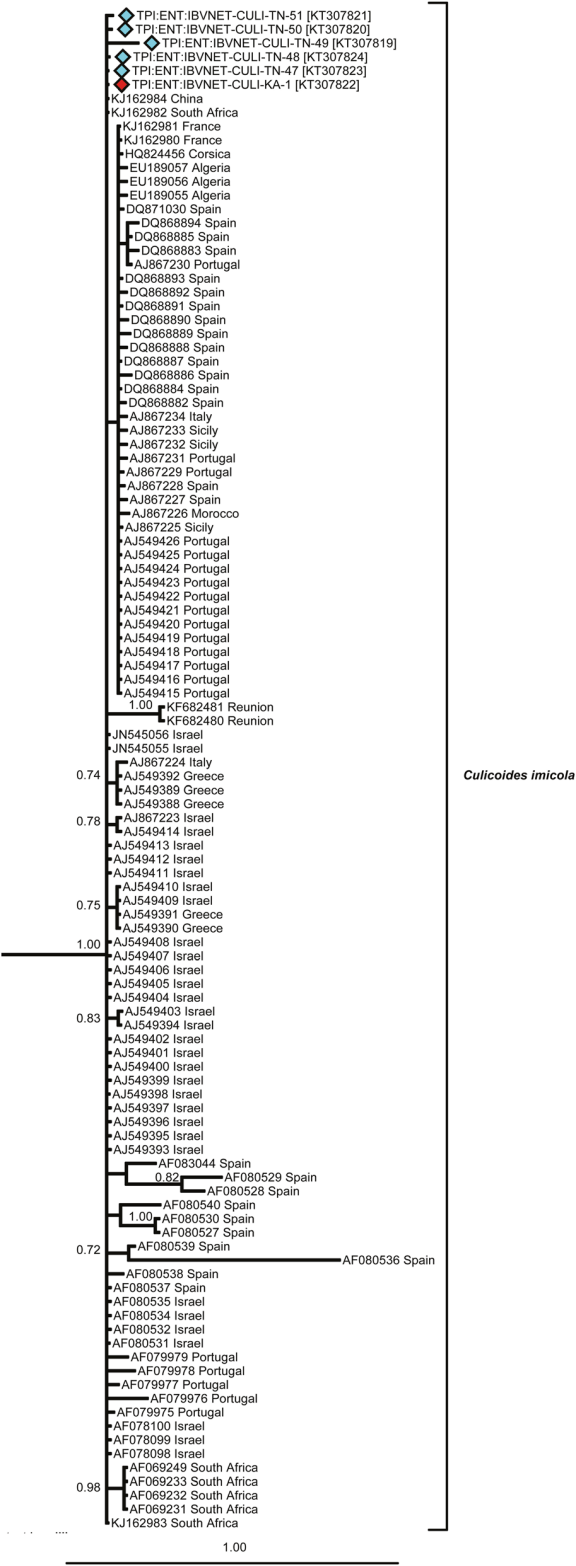
Bayesian Inference phylogenetic tree of the *C. imicola* clade inferred from *COI* DNA barcode sequences. Bayesian posterior probability node support values greater than 0.7 shown. Coloured diamonds indicates specimens from the IBVNet project coloured by collection site (KA01: *red*; TN01: *purple*; TN02: *green*; TN08: *blue*; TN10: *orange*; TN11: *yellow*; TN12: *black*) with specimen number followed by GenBank accession number in brackets. See Fig. 4 for the relative placement of the *C. imicola* clade with respect to other specimens analysed within this study

**Fig. 6:**
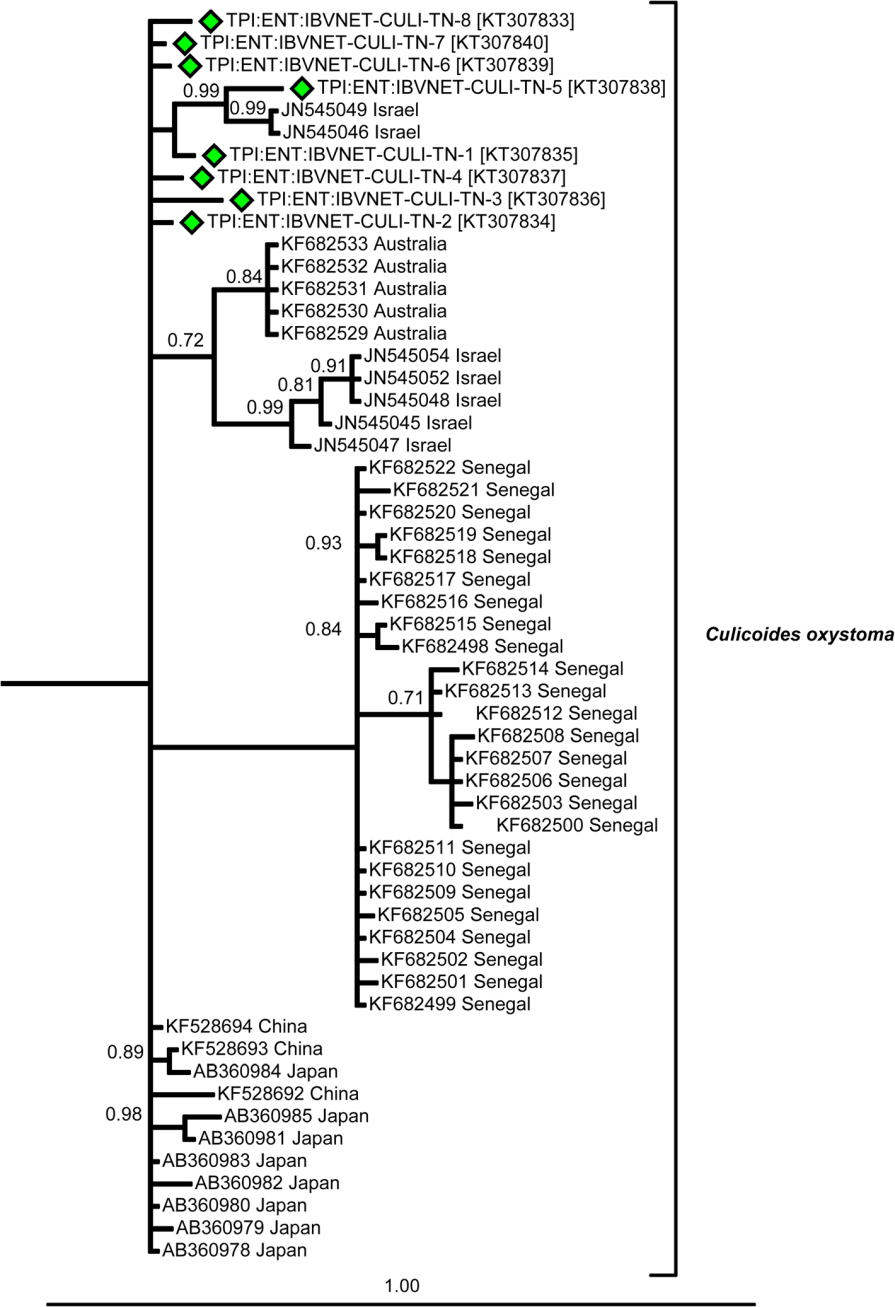
Bayesian Inference phylogenetic tree of the *C. oxystoma* clade inferred from *COI* DNA barcode sequences. Bayesian posterior probability node support values greater than 0.7 shown. Coloured diamonds indicates specimens from the IBVNet project coloured by collection site (KA1: *red*; TN01: *purple*; TN02: *green*; TN08: *blue*; TN10: *orange*; TN11: *yellow*; TN12: *black*) with specimen number followed by GenBank accession number in brackets. See Fig. 4 for the relative placement of the *C. oxystoma* clade with respect to other specimens analysed within this study

**Fig. 7:**
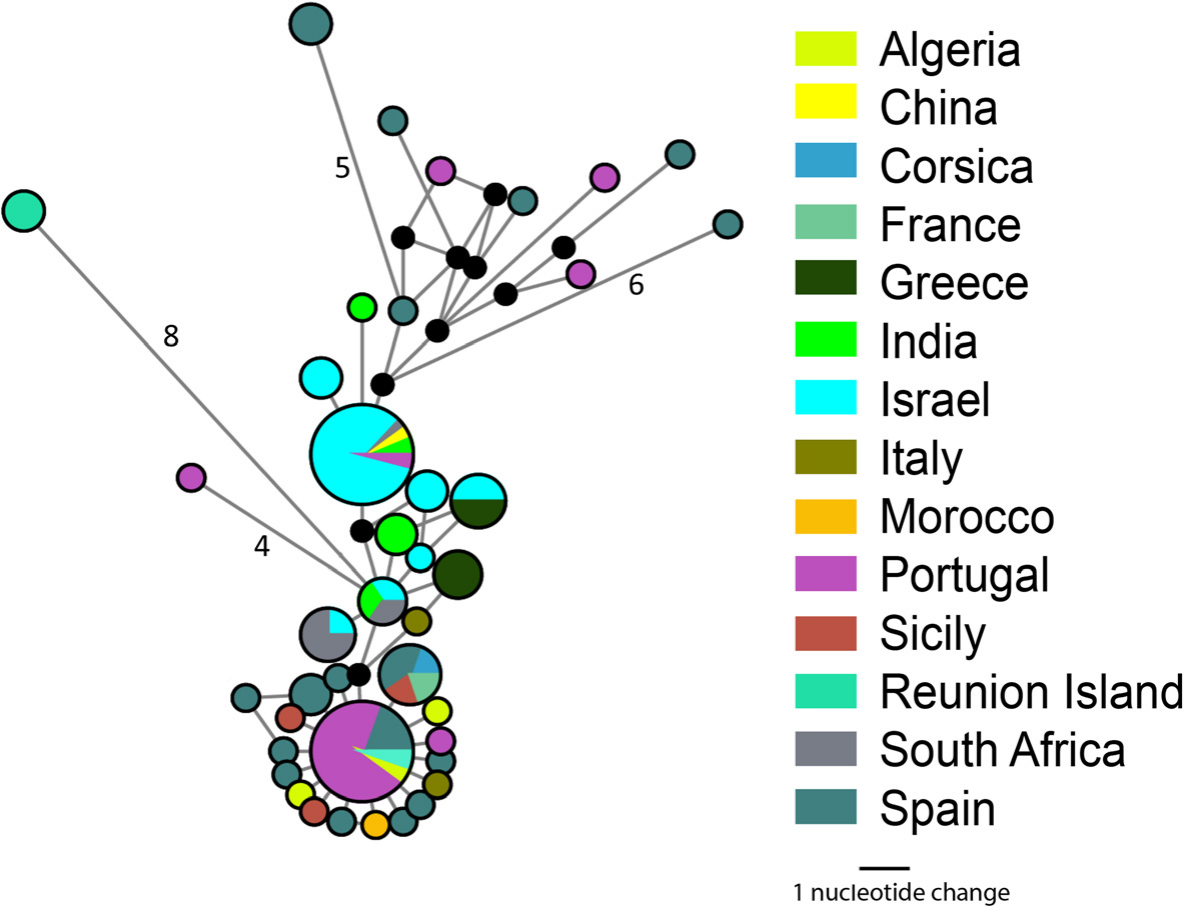
Most parsimonious Median-Joining Network (ε = 0) depicting the phylogenetic relationships among, and geographical assignment, of *C. imicola COI* haplotypes. The size of each circle is proportional to the corresponding haplotype frequency. Branch lengths are proportional to the number of nucleotide changes between haplotypes. *Black circles* indicate median vectors (*mv*) that represent hypothetical missing or unsampled ancestral haplotypes. Number of nucleotide changes indicated on longer branches (Sequences AF080528, AF080529 and AF080536 excluded from analysis)

**Fig. 8:**
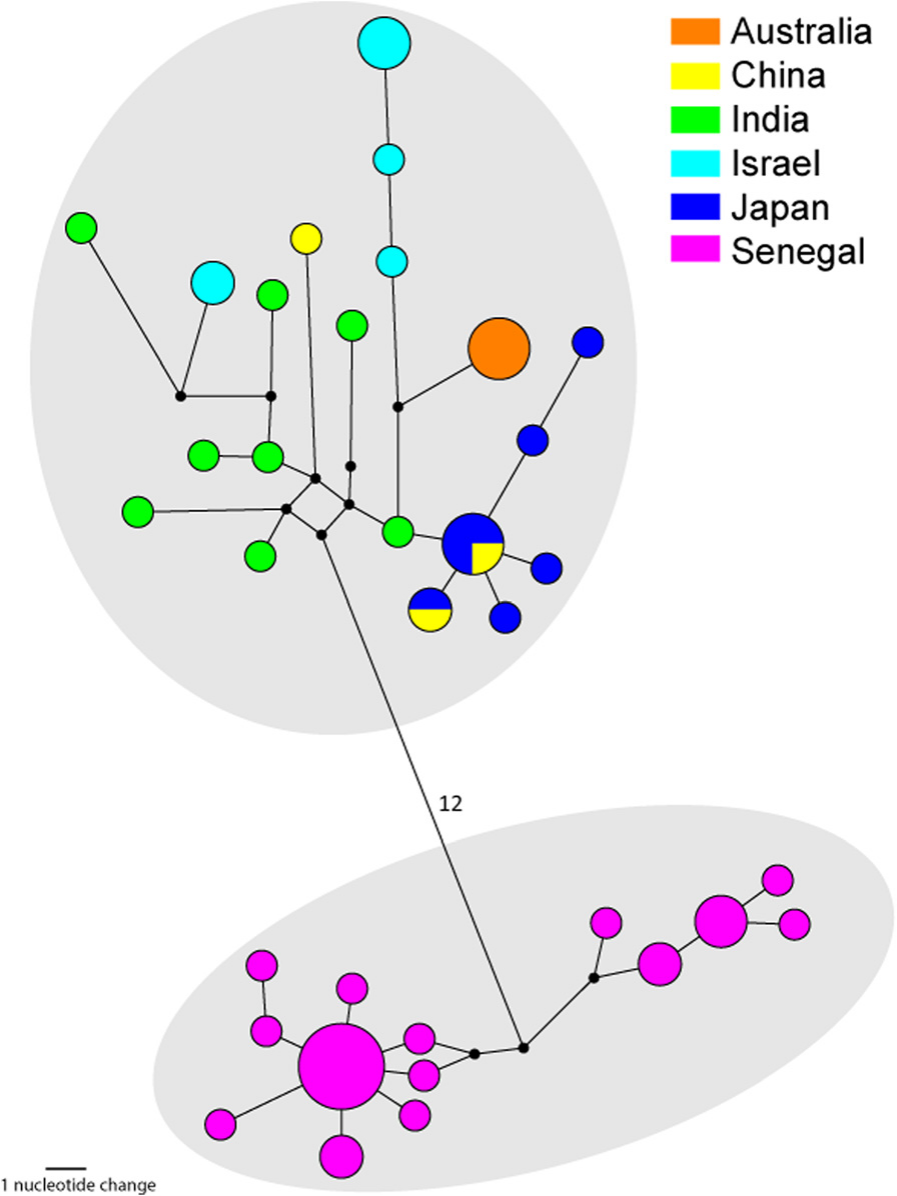
Most parsimonious Median-Joining Network (ε = 0) depicting the phylogenetic relationships among, and geographical assignment, of *C. oxystoma COI* haplotypes. The size of each circle is proportional to the corresponding haplotype frequency. Branch lengths are proportional to the number of nucleotide changes between haplotypes. *Black circles* indicate median vectors (*mv*) that represent hypothetical missing or unsampled ancestral haplotypes. Number of nucleotide changes indicated on longer branches

**Fig. 9:**
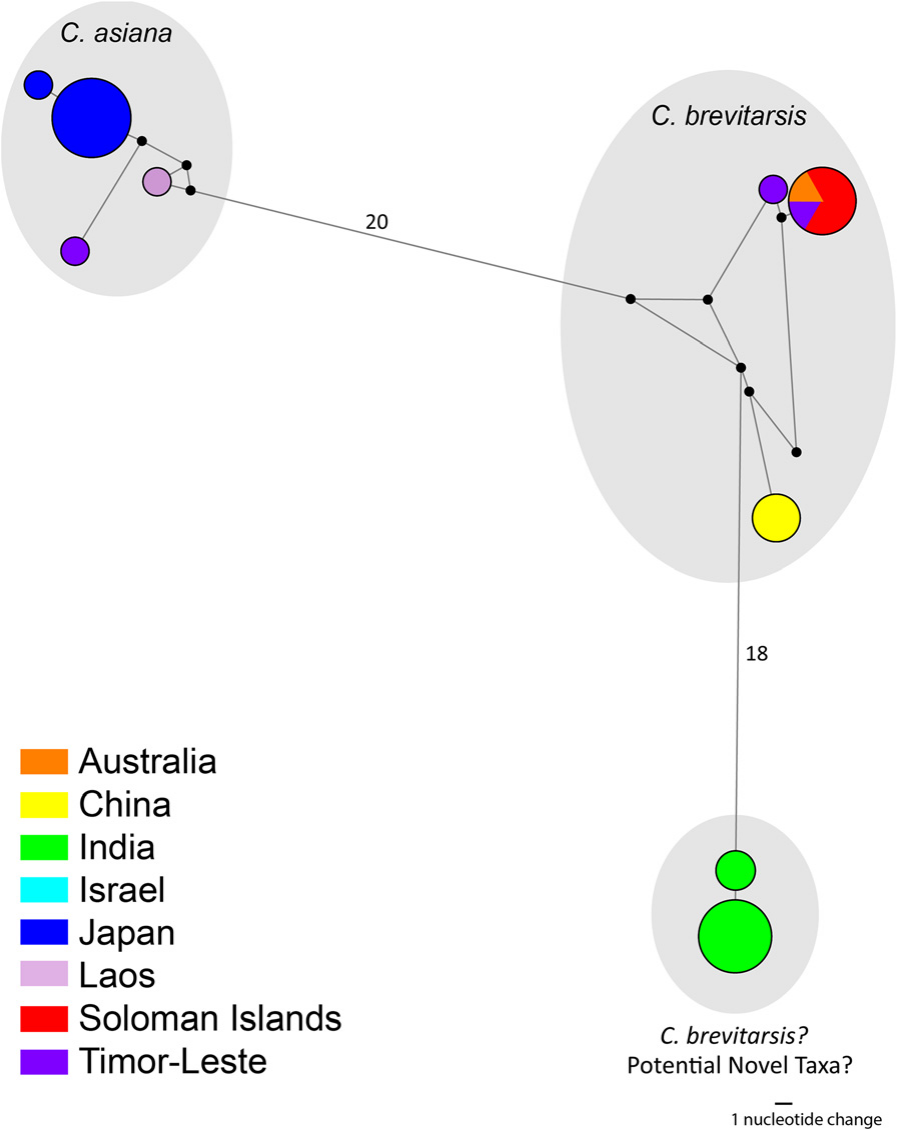
Most parsimonious Median-Joining Network (ε = 0) depicting the phylogenetic relationships among, and geographical assignment, of C. *brevitarsis* and *C. asiana COI* haplotypes. The size of each circle is proportional to the corresponding haplotype frequency. Branch lengths are proportional to the number of nucleotide changes between haplotypes. *Black circles* indicate median vectors (*mv*) that represent hypothetical missing or unsampled ancestral haplotypes. Number of nucleotide changes indicated on longer branches

Thirteen species from the collections in this study and a further six potentially morphologically confounding species (19 in total) were represented within the phylogenetic study (Figs. 4, 5, 6, 7, 8 and 9). Species clades represented in the Bayesian Inference (BI) phylogeny were concordant with morphological identifications with the exception of one specimen, TPI:ENT:IBVNET-CULI-TN-65, which could not be assigned to a species based on the morphological descriptions currently available in the literature, and is therefore recorded as ‘Unknown Species (I)’ (Fig. 4, Additional file 1: Table S2 and Additional file 2: Table S3). No discordant Barcode Index Numbers (BINs) [71] were observed for the species identifications of specimens collected within this study, with the exception of *C. huffi,* which resulted in two BINs being assigned thereby indicating the potential presence of two distinct taxa (see Additional file 1: Table S2). In addition, *C. actoni*, *C. brevitarsis*, *C. imicola* and *C. peregrinus* currently all have multiple BINs per species name currently assigned within BOLD [44], indicating the presence of either misidentified specimens or unresolved cryptic diversity within the publically avaliable data.

**Fig. 10:**
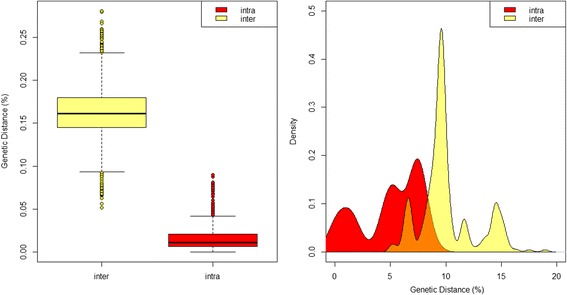
Box-and-whisker plots (*left*) and frequency distribution plot (*right*) of interspecific, i.e. closest non-conspecific (*yellow*) and intraspecific, i.e. the furthest intraspecific distance among its own species (*red*) pairwise genetic distances (uncorrected percentage sequence distances) across all species in this study. Areas where the intra- and inter-specific distances overlap shown in *orange*

**Fig. 11:**
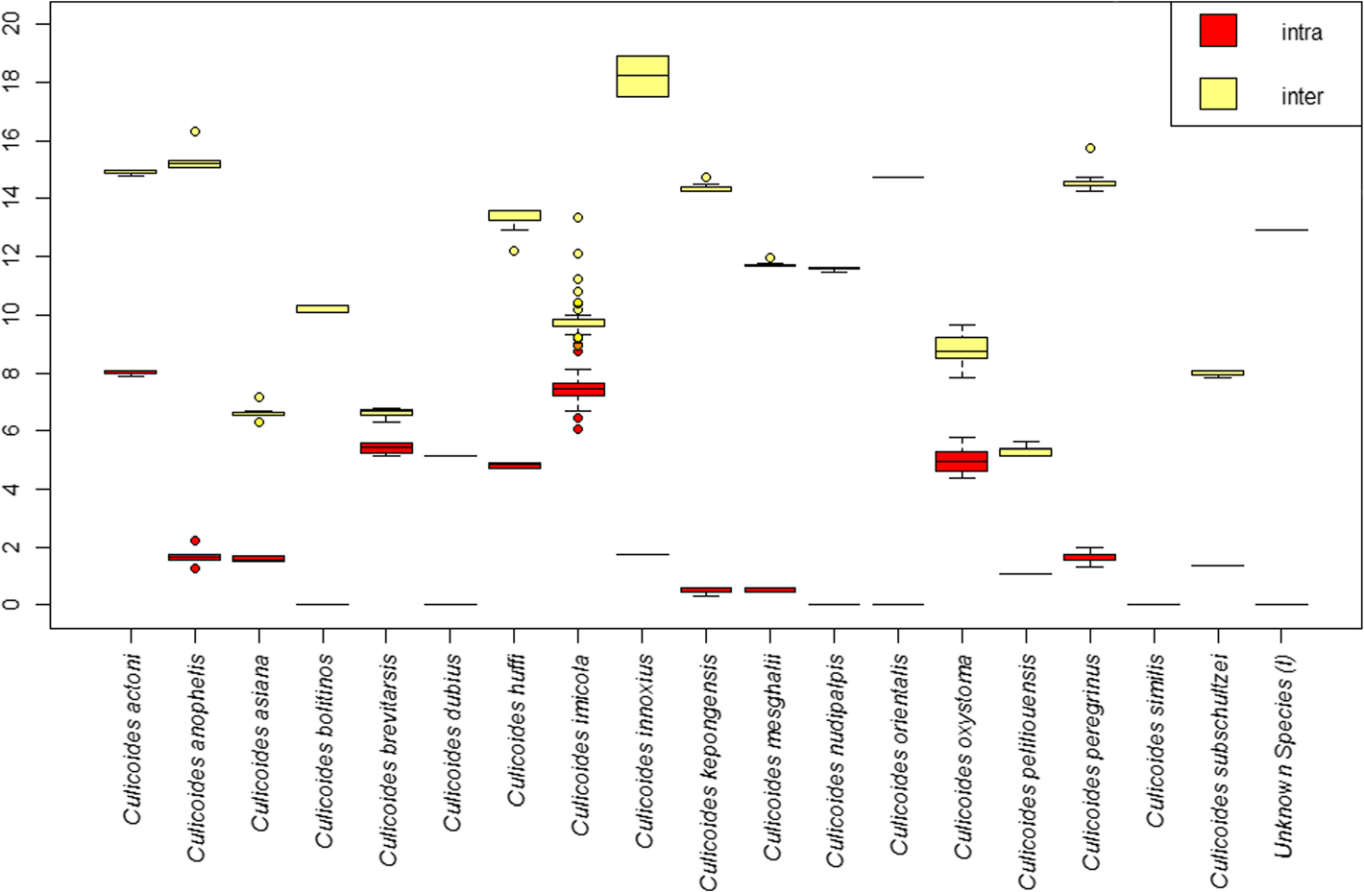
Box-and-whisker plots of the interspecific, i.e. closest non-conspecific (*yellow*) and intraspecific, i.e. the furthest intraspecific distance among its own species (*red*) pairwise genetic distances (uncorrected percentage sequence distances) by species. Areas where the intra- and inter-specific distances overlap shown in *orange*

Deep interspecific differences within the *COI* DNA barcode region were present between the majority of *Culicoides* assessed within this study; however, there was no clear barcoding gap [72, 73] across all current species assignments (Fig. 10). The greatest intraspecific sequence differences were reported from within specimens morphologically identified as *C. actoni* (mean: 5.8 %; range: 1.5–8.1 %), followed by *C. brevitarsis* (mean: 3.2 %; range: 0–5.6 %), *C. huffi* (mean: 2.5 %; range: 0–4.9 %), *C. imicola* (mean: 1.2 %; range: 0–9.0 %) and *C. oxystoma* (mean: 2.9 %; range: 0–5.8 %) (Additional file 2: Table S3; Fig. 11). These levels of sequence variation is more akin to interspecific values (Additional file 2, Table S3), indicating cryptic taxa in the samples sequenced within these taxa, or morphological misidentifications in our dataset. All other pairwise intraspecific sequence differences were less than or equal to 2.0 %, i.e. within published ranges of intraspecific variation [74] (Additional file 2: Table S3; Fig. 11). The least mean sequence difference was between *C. dubius* Arnaud, 1956 and *C. peliliouensis* (mean: 5.3 %; range: 5.2–5.6 %); *C. asiana* and *C. brevitarsis* (mean: 7.0 %; range: 6.3–7.9 %), and *C. oxystoma* and *C. subschultzei* Cornet & Brunhes, 1994 (mean: 9.1 %; range: 7.8–10.4 %) and *C. brevitarsis* and *C. imicola* (mean: 10.7 %; range: 9.0–15.0 %) (Additional file 2: Table S3). All other pairwise interspecific sequence difference were greater than 10.0 % (Additional file 2: Table S3). No misidentifications occurred between species identified within the study and those considered isomorphic or morphologically similar for which *COI* DNA barcode sequence data was available.

No geographic clustering was observed in the *C. imicola* haplotypes (Figs. 5 and 7). Three specimens collected in previous studies from Spain (GenBank: AF080528, AF080529 and AF080536) were, however, found to have between 2.7 and 4.6 %, 3.5–5.4 % and 6.0–9.0 % sequence difference, respectively, to other specimens identified as *C. imicola*. The range of intraspecific sequence differences within *C. imicola* when these three sequences were excluded was significantly reduced from mean of 1.2 % (range: 0–9.0 %) to mean of 1.0 % (range: 0–3.9 %). Sequences AF080528, AF080529 and AF080536 are likely to be either misidentifications or poor quality sequences and were excluded from subsequent investigations of haplotype relationships within *C. imicola.*

A significant degree of geographic clustering was observed in the *C. oxystoma* haplotypes (Figs. 6 and 8), with haplotypes previously recorded from Senegal [9] clustering separately from specimens collected in the current study and from those in Australia, China, India, Israel and Japan [75, 76] which formed an Oriental-Australasian clade with between 3.7 and 5.8 % (mean: 4.3 %) sequence difference between the Oriental-Australasian and Senegalese clades (Fig. 8).

Specimens morphologically identified as *C. brevitarsis* collected in southern India were strongly supported by BI as a monophyletic clade (100 % posterior probability) with sequence difference between 5.0–5.6 % (mean: 5.3 %) (Fig. 9) to specimens collected from Australia, China, the Solomon Islands and Timor-Leste [26]. They also differed from *C. asiana* specimens from Japan (= *C. brevitarsis* [75], misidentification [26]) (mean sequence difference: 6.9 %; range: 6.5–7.3 %) (Fig. 9).

The three *C. actoni* sequences demonstrated 8.1 and 1.5 % sequence difference between the specimen collected in this study from southern India and the sequences previous published from Australia [26] and Japan [75], respectively. In comparison, Japanese and Australian *C. actoni* specimens showed 7.9 % sequence difference.

### Green *versus* UV wavelength comparison trial

A total of 7284 *Culicoides* were collected using the green LED CDC trap, while an estimated total of 120,460 individuals were collected using the UV LED CDC trap. Both trap types collected *Culicoides* on each day of the trial, with a maximum estimated single night catch of 13,022 individuals (trap night seven) in the UV LED CDC trap, and 650 individuals (trap night 14) in the green LED CDC trap. Trap catches were heavily biased towards female specimens in both the UV LED CDC trap (90.9 %) and the green LED CDC trap (89.0 %) collections (Table 1). Non-pigmented females dominated the collections in both trap types (51.9 % UV; 48.3 % green), followed by blood-fed (20.4 % UV; 21.8 % green) and pigmented individuals (18.0 % UV; 18.5 % green), with only a few gravid females collected (0.6 % UV; 0.4 % green) (Table 1). The relative proportion of the trap catches made up of the different parity states were not significantly different between the UV and green LED CDC traps; however, the UV LED CDC trap collected significantly more male and female *Culicoides* than the green LED CDC trap (Paired *t*-test: *t* = 6.464, *df* = 19, *P* < 0.001). Over 16 times more females were collected using the UV LED CDC trap than in the green LED CDC trap.

**Table 1:** *Culicoides* spp. collected during 20 nights comparative trapping in at site TN01 using light emitting diode (LED) Center for Disease Control (CDC) light-suction traps (mean number collected per night with range shown in parentheses

Species	Green LED CDC trap (~570 nm peak wavelength)							UV LED CDC trap (~390 nm peak wavelength)^a^						
	Np^b^	Bf^b^	G^b^	P^b^	M^b^	Total catch	Appearances^c^	Np^b^	Bf^b^	G^b^	P^b^	M^b^	Total catch	Appearances^c^
*C. anophelis*	9.5 (0–23)	0.1 (0–1)	0.1 (0–1)	0.1 (0–1)	0.2 (0–2)	9.7 (0–23)	19	99.0 (0–341)	4.6 (0–50)	4.7 (0–35)	–	1.1 (0–14)	5.5 (0–99)	19
*C. brevitarsis*	0.3 (0–2)	0.2 (0–1)	–	0.2 (0–1)	–	0.7 (0–2)	8	16.8 (0–122)	1.3 (0–11)	0.1 (0–1)	8.1 (0–74)	2.8 (0–25)	1.5 (0–122)	15
*C. huffi*	–	–	–	–	–	–	–	–	–	0.2 (0–5)	–	–	0.2 (0–5)	1
*C. imicola*	0.1 (0–4)	0.3 (0–1)	–	0.3 (0–2)	1.5 (0–14)	3.0 (0–14)	17	29.9 (0–136)	7.6 (0–63)	–	10.9 (0–84)	14.8 (0–52)	63.1 (0–136)	19
*C. innoxius*	0.1 (0–1)	–	–	–	–	0.1 (0–1)	1	0.3 (0–7)	0.4 (0–9)	–	0.9 (0–17)	–	5.3 (0–23)	8
*C. oxystoma*	156.3 (2–317)	45.6 (4–105)	1.2 (0–6)	60.5 (4–120)	37.6 (8–117)	301.2 (0–317)	20	2,537.5 (262–8,034)	248.3 (29–499)	12.9 (0–101)	608.1 (87–1,380)	473.5 (46–1,102)	3,880.3 (0–8,034)	20
*C. peliliouensis*	–	0.1 (0–1)	–	–	–	0.1 (0–1)	1	2.3 (0–31)	0.5 (0–10)	–	–	–	0.1 (0–31)	3
*C. peregrinus*	8.7 (0–27)	33.1 (4–95)	0.4 (0–4)	6.4 (0–22)	0.8 (0–4)	49.4 (0–95)	20	437.4 (65–1,898)	964.1 (66–3,265)	19.8 (0–175)	454 (15–2,366)	57.6 (0–452)	1,932.8 (0–3,265)	20

*Abbreviations*: *Np* non-pigmented females, *Bf* blood-fed females, *G* gravid females, *P* pigmented females, *M* males

^a^Estimated using standardised subsampling procedure [66]

^b^-dash indicates species/ sex/ parity state not collected

^c^number of nights out of 20

**Fig. 12:**
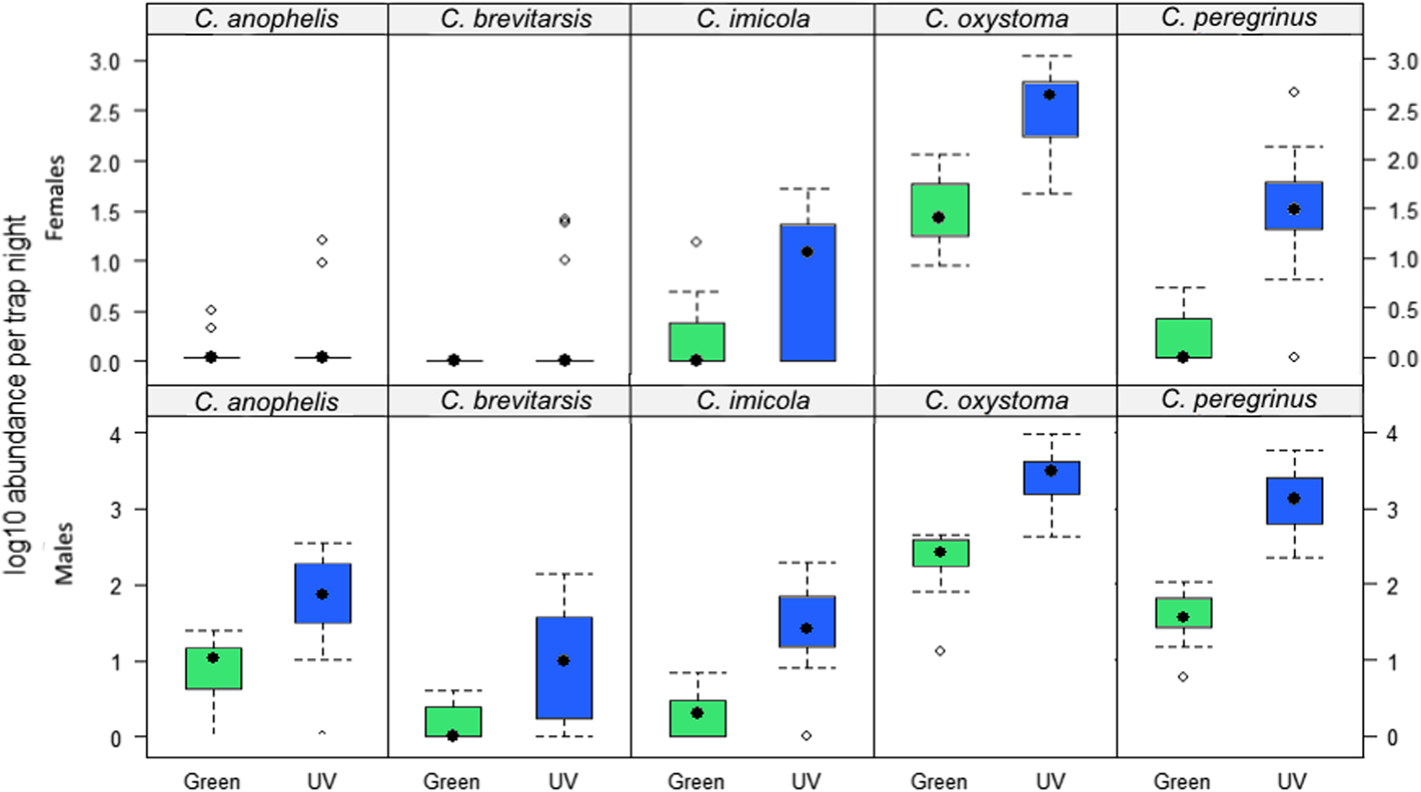
Log_10_ median abundance of the specimens collected per trap night for the five most abundant *Culicoides* spp. collected by green light emitting diode (LED) Center for Disease Control (CDC) traps as compared to ultraviolet (UV) LED CDC traps, stratified by species and sex

Eight species (morphologically identified) were represented within the LED CDC trap collections (in order of abundance): *C. oxystoma*, *C. peregrinus*, *C. imicola*, *C. brevitarsis*, *C. anophelis*, *C. innoxius, C. peliliouensis* and *C. huffi*. All species were present in both UV and green LED CDC trap collections, with the exception of *C. huffi,* which was absent from green LED CDC trap collections (Table 1). Trap catches from both trap types were dominated by *C. oxystoma* and *C. peregrinus* which together represented 65.6 and 32.7 % of *Culicoides* specimens in the UV, and 82.7 and 13.6 % of *Culicoides* specimens in the green trap collections respectively, with the other species collected making up just 3.7 and 1.8 % of the trap catch collectively for the green and the UV trap, respectively (Table 1, Fig. 12).

**Fig. 13:**
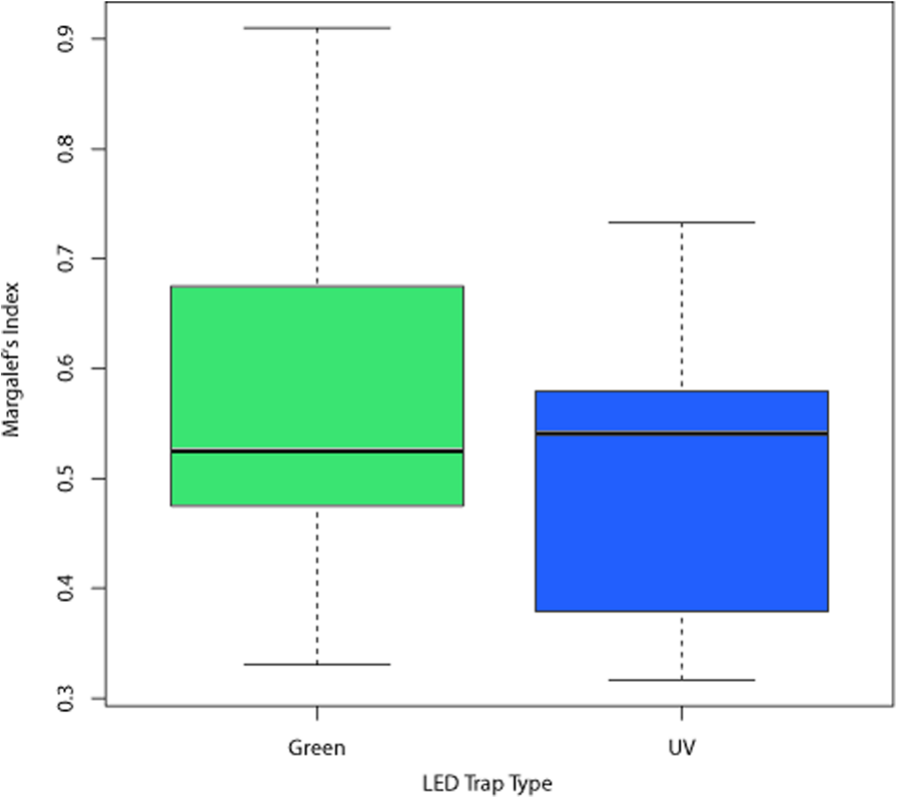
Box-and-whisker plots of Margalef’s Index illustrating the relative variability in species richness in relation to collection by the ultraviolet (UV) compared to green light emitting diode (LED) Center for Disease Control (CDC) trap

The relative abundance of the most abundant species, *C. oxystoma* and *C. peregrinus*, significantly varied between the green and UV traps (Fig. 12). Overall diversity (i.e. the number of different *Culicoides* spp. present within a trap catch) did not vary significantly between the green and UV LED CDC traps, as measured by the Margalef’s index (mean: 0.50; range: 0.32–0.73 and mean: 0.57; range: 0.33–0.91 for the UV and green LED CDC traps, respectively) (Fig. 13).

Due to the low numbers collected, *C. innoxius, C. huffi* and *C. peliliouensis* were excluded from the following statistical analysis. Numbers of female *Culicoides* collected by the LED CDC traps were significantly affected by the variables *species* and *light type* and the by the interaction variable *species* × *light type* (Table 2). The UV light trap collected significantly more female *C. peregrinus* than the green LED CDC trap. There were, however, no significant differences in the numbers of female *C. anophelis*, *C. brevitarsis*, *C. imicola* or *C. oxystoma* collected by the UV compared to green LED CDC trap (Table 2; Fig. 12). *Species* and *light type* were also key determinants of the numbers of male *Culicoides* collected (Table 2). The UV LED CDC traps collected a significantly larger number of male *C. imicola*, *C. oxystoma* and *C. peregrinus* than the green LED CDC traps (Table 2; Fig. 12). There were, however, no significant differences in the numbers of male *C. anophelis* and *C. brevitarsis,* collected by the UV compared to green LED CDC trap (Table 2; Fig. 12).

**Table 2:** Regression coefficients with standard errors (SE) for the fixed effects of the two final general linear mixed models with (i) a binomial error distribution for the total number of female *Culicoides* spp. collected and (ii) a negative binomial error distribution for the total number of male *Culicoides* spp. collected

Parameter		Total female *Culicoides* spp. collected		Total male *Culicoides* spp. collected	
		Estimate	SE	Estimate	SE
Intercept		0.45	0.35	-2.18***	0.53
Light Type	UV	3.55***	0.38	0.89***	0.21
Species	*C. imicola*	0.77	0.42	2.60***	0.54
	*C. oxystoma*	6.05***	0.38	5.75***	0.54
	*C. peregrinus*	4.33***	0.38	2.87***	0.51
	*C. anophelis*	2.64***	0.39	0.05	0.60
Position	Position 2	–	–	-0.19	0.65
Species × Light Type	*C. imicola* : UV	0.16	0.51	–	–
	*C. oxystoma* : UV	-1.11	0.46	–	–
	*C. peregrinus* : UV	-0.07	0.46	–	–
	*C. anophelis* : UV	-1.16	0.47	–	–
Species × Position	*C. imicola* : Position 2	–	–	-0.34	0.75
	*C. oxystoma* : Position 2	–	–	-0.04	0.71
	*C. peregrinus* : Position 2	–	–	0.22	0.72
	*C. anophelis* : Position 2	–	–	-2.82	1.32

***P ≤ 0.001

Random effects included in the final models included the effect of trapping day. N-dash indicates variable not included in model

## Discussion

This study presents the first detailed multi-site genetic analysis of *Culicoides* in southern India with new *COI* DNA barcode sequence data presented for 12 previously recognised species, and potentially up to four cryptic or unidentified taxa. To the best of our knowledge, *Culicoides mesghalii* and *C. kepongensis* are recorded for the first time in India and *C. peliliouensis* and *C. similis* are recorded for the first time in southern India. The study also reveals the potential for deep sequences differences symptomatic of cryptic species diversity within *C. actoni*, *C. brevitarsis* and *C. huffi*, which require further investigation. In addition, further evidence of geographic clustering and potential cryptic species diversity within *C. oxystoma* is presented*,* in support of that identified by Bakhoum et al. [9]. With these caveats, morphological identification of the species considered the most likely vectors of BTV in India was demonstrated to be robust, with no misidentifications of the main putative vector species. In addition, commercially produced UV LED light-suction traps were shown to outperform traps fitted with green LED’s for *Culicoides* spp. collection, based on both the total number and diversity of specimens of *Culicoides* collected. These findings collectively address many of the logistical requirements for effective *Culicoides* spp. sampling in southern India.

The provision of DNA barcode data for putative BTV vector species in southern India fills an important gap in our knowledge of the phylogeny of these species. An east–west split in haplotypes of *C. imicola* specimens from the Mediterranean basin was previously identified using *COI* sequencing by Calvo et al. [77], Dallas et al. [78] and Nolan et al. [79]. The present study has shown that four *C. imicola COI* haplotypes are present in southern India; two are unique to India, one is identical to specimens collected in Israel and South Africa and the other is identical to specimens collected in China, Israel, South Africa and Portugal. The status of the latter sample from Portugal (GenBank: AF079975) requires re-evaluation as this sample does not fit with previously proposed eastern-western haplotype demarcation in the Mediterranean basin. In addition, three publically available sequences (GenBank: AF080528, AF080529 and AF080536) labelled as *C. imicola* but with sequences differences of 2.9, 3.7 and 7.5 % respectively to the nearest other *C. imicola* sequence have been highlighted as likely to be the result of misidentification, cross-contamination or poor sequence quality. Genetic divergence and population structure within *C. imicola*, the principal Afrotropical vector of BTV, are subject to on-going investigations [80, 81] that will assist in clarifying the status of this pan-continental species.

The status of the subgenus *Remmia* Glukhova (= Schutzei group) of *Culicoides* in Asia has historically been fraught with confusion [18]. This is particularly the case in India with numerous publications citing the presence of *C. schultzei* [12–14, 81–86], despite the fact that *C. schultzei* is an Afrotropical species while the type-locality of *C. oxystoma* is Kolkata in India. These two species are, however, both morphologically [18, 87] and genetically [9, 76, 88] distinct, and current evidence agrees with the earlier proposal by Cornet [89] that *C. oxystoma* is the species present in India. Furthermore, Indian *C. oxystoma* specimens form part of the Saharo-Arabian-Oriental-Australian clade, but further investigation is needed to see if the two genetic groupings of Bakhoum et al. [9] correlate with the morphological variation observed by Wirth & Hubert [18], and whether they are supported by data from additional genetic markers.

Recent re-evaluations of the Imicola Complex by Bellis et al. [26] provide strong evidence that *C. brevitarsis* specimens identified in Japan are in fact the novel species *C. asiana* [26] (= *C. asiatica* Bellis, 2014 [54]). Further cryptic diversity between Australian and Chinese *C. brevitarsis* was suggested by analysis of the *COI* region, but this was not supported by analysis of the fused carbamoyl phosphate synthetase, aspartate transcarbamylase and dihydroorotase (CAD) nuclear genes [26]. Similar investigations are required to explore the potential cryptic diversity identified between the Indian and Australian specimens of *C. brevitarsis*. There is no evidence that the morphologically indistinguishable *C. bolitinos* [26, 90] is present in southern India as this species is genetically distinct from the Indian *C. brevitarsis* (mean sequence difference compared to South African *C. bolitinos*: 12.2 %; range: 11.6–12.6 %)*.* The Indian *C. brevitarsis* is also genetically distinct (mean sequence difference: 11.1 %; range: 10.6–11.5 %) from *C. bolitinos* specimens recently collected in Reunion Island (GenBank: KF186129 and KF186130) [91], which have been provisionally identified as *C. bolitinos*, but which have been shown to be genetically distinct from South African *C. bolitinos* (mean sequence difference: 6.4 %; range 5.6–6.9 %). The latter relationship and the implications of this potential cryptic diversity within *C. bolitinos* are yet to be resolved.

The single *C. actoni COI* sequence from India is consistent with the Asian clade of this species, and is consequently different to the species in Australia [92] upon which vector competence studies for this species are based [93]. As such, there is no data on the vector potential of Indian *C. actoni*. Nevertheless, the Indian species, which almost certainly belongs to *C. actoni* (*senso stricto*) [92], is closely related to the proven vector in Australia and warrants investigation into its potential as a vector of BTV.

The sequence difference between *C. peregrinus* specimens from China, Japan, India and Thailand was 1.2–1.7 %. However, Australian specimens have been the basis of *C. peregrinus* BTV vector incrimination studies [93] and the south Indian specimens of *C. peregrinus* assessed within this study show only limited sequence divergence (mean sequence difference: 2.0 % (1.8–2.3 %) from specimens recently collected in Australia (GenBank: KR075719–KR075721) [94], indicating these associations are intraspecific and are still valid.

*Culicoides anophelis* has previously been reported from southern India [11, 12, 95] and is widespread throughout southern Asia [18] possibly ranging as far east as New Guinea [96]. The sequence identity of *C. anophelis* specimens collected in this study were concordant with the only available *COI* sequence for this species, also from southern India, and further study is required to investigate the relationship of populations of this species across its extensive range. *Culicoides innoxius* is widely distributed across southern Asia [18, 97], including Bangalore in southern India [11]. This species is very similar morphologically to *C. sumatrae* Macfie, 1934 [18], but there is no molecular sequence data currently available for *C. sumatrae* so we are not able to test the validity of these species. Similarly, comparisons between *C. innoxius* and other Indian species of the subgenus *Hoffmania* of *Culicoides* (for example those described by Majumdar et al. [39]), would help clarify the status of these species in India.

*Culicoides mesghalii* and *C. kepongensi*s are recorded for the first time in India and *C. peliliouensis* and *C. similis* for the first time in southern India, the latter having previously been recorded in West Bengal [98] and West Bengal and Jharkhand [40], respectively. This study provides the first sequence data (*COI* DNA barcode) for these species. *Culicoides mesghalii* is known to occur in the Saharo-Arabian region [38], *C. peliliouensis* in the Oriental region [18, 97] and *C. similis* from across the Afrotropical [99, 100], Saharo-Arabian [38] and Oriental [18, 101] regions, so the presence of these species in southern India is not surprising and probably reflects a paucity of collecting in southern India, rather than recent incursions. The specimens within this study identified as *C. similis* are consistent with the description of this species given by Nandi & Mazumdar [40]. The morphological description of *C. similis* by Nandi & Mazumdar [40] is, however, in contrast to that given for *C. similis* specimens from across the Afrotropical [99, 100], Saharo-Arabian [38] and Oriental [18, 101] regions. The specimens collected in this study have therefore tentatively been recorded as *C. similis,* however, the variation in diagnostic morphological characters for this species in the literature warrants further investigation to confirm the validity of this identification and whether the *C. similis* described by Nandi & Mazumdar [40] in fact represents a novel species or simply a morphological variant of *C. similis. Culicoides peliliouensis* is also considered to be morphologically similar to the Cambodian species *C. pongsomiensis* Chu, 1986 [18]. Collection of additional specimens and generation of DNA barcodes for the latter species would allow the exploration of the validity or potential synonymy of these taxa.

The *C. huffi* specimens identified in this study are consistent with the morphological description of this species by Wirth & Hubert [18]. Sequences differences [mean: 4.7 % range: 4.4–4.9 %)] were noted between *C. huffi* specimens collected in Karnataka and those collected in Tamil Nadu (Fig. 4). Variation has been noted in the morphological descriptions provided for *C. huffi* by Nandi & Mazumdar [40] and Wirth & Hubert [18], however further specimens are required to clarify the status of this species in India, and compare these with topotypic specimens from Thailand [102]. Processing additional *C. huffi* specimens from a wider geographic area in India and specimens from morphologically similar species including *C. palpisimilis* Wirth & Hubert, 1989 and *C. similis* [18] would further resolve the delineation of these species and aid in resolving their subgeneric placement.

This study provides further evidence of the potential utility of DNA barcodes for species identification within *Culicoides*, and its potential to identify areas of potential cryptic diversity, which require further investigation, e.g. *C. actoni* [92]. This utility is, however, proportional to the number of species represented within the reference dataset and a strong integration with morphological taxonomy [28]. Further DNA barcode data is also required across the full range of the *Culicoides* subgenera in order to test the ‘barcode gap’ hypothesis related to the definition of species boundaries with regard to intra- and inter-specific variation [72]. The current subgeneric classification of the *Culicoides* [103], however, remains largely unvalidated (for review see Harrup et al. [28]) and there is mounting evidence to suggest that at least some of the current subgenera are polyphyletic, i.e. derived from more than one common ancestor [104–107]. The additional *Avaritia* subgenus species sequence data presented within this study, however, supports the monophyly of this subgenus within the context of the species included in the phylogenetic analysis with the *Avaritia* subgenus supported by BI as a monophyletic clade (100 % posterior probability).

The UV LED-based light-suction traps tested in this study clearly outperformed the alternative green LED model in the number of *Culicoides* collected, with more than 16 times the number of individuals collected. Collections of *C. brevitarsis,* predicted to be under-represented in UV-based light-suction trap collections based on previous studies in Australia [31], were in fact collected in greater numbers in the UV LED-based trap compared to the green LED-based trap, indicating that a UV-based trap is sufficiently sensitive to collect *C. brevitarsis* in southern India. Also of significant importance is the absence of key potential BTV vector species, including *C. imicola* and *C. peregrinus,* on multiple nights from green LED-based light-suction trap collections when these species are present on the corresponding night in the UV LED-based light-suction trap collection. If this were to occur during surveillance activities, the reduced sensitivity of the green LED-based light-suction traps would result in pseudo-absences within the *Culicoides* abundance dataset potentially resulting in epidemiologically significant errors in models of *Culicoides* seasonality and/or distribution. Ultraviolet LED-based light-suction traps, however, did collect a greater by-catch of other insects in comparison to green LED-based traps and hence incurred a greater handling time for each sample collected. While taking into account the limited spatial-scale and temporal duration of this comparison of green and UV wavelength-based light-suction traps the increased sensitivity of detection of the UV LED-based trap makes it the preferred choice for on-going surveillance efforts in India compared to a green LED-based light-suction trap.

Ultraviolet-based light-suction traps have previously been recommended as the gold-standard for collecting *Culicoides* [108], based initially on the Onderstepoort Veterinary Institute (OVI) trap design. Following on from the recommendations of Mellor et al. [108], multiple UV-based light-suction traps have been utilised for the collection of *Culicoides* including those based on conventional fluorescent UV bulbs and LEDs [109, 110] with the numbers of *Culicoides* collected by the traps roughly proportional to the intensity of the UV light emitted by the trap and the power of the fan. While the LED CDC type trap tested in this study has previously been found to be outperformed by CDC type traps fitted with a conventional fluorescent UV bulb [109], this variation is likely due to variation in the light intensity between the traps. However, the significantly lower power consumption and weight of the LED-based traps in comparison to currently available fluorescent bulb-based traps increases convenience for collectors and increases the number of nights of collections which can be completed on one battery charge from one to four nights when used with a ≥10 Ah battery and photo switch. The increased power efficiency of LED-based light-suction traps in comparison to conventional fluorescent UV bulb-based light-suction traps, such as the OVI, may assist in establishing *Culicoides* trapping schemes in areas where there are significant logistical challenges such as limited access, limited mains electricity availability, and financial constraints with regard to the purchasing of additional batteries [35, 36].

A major consideration for establishing light-suction trapping networks is their limitations in reflecting the abundance and diversity of *Culicoides* feeding on ruminants. Diurnal activity has been reported in *C. oxystoma* [111] and *C. actoni* [18] indicating that any trap utilising light as an attractant has the potential to underestimate the abundance and distribution of these species in comparison to species with a principally crepuscular or nocturnal activity pattern. In addition, the preferential attraction to different light wavelength of the highly competent BTV vector *C. fulvus,* which is known from northern [1, 5, 6], but not southern India, remains unknown as it was not detected during this study. Further studies across different environments in this region would be useful in elucidating the distribution of this and other putative BTV vectors in this region. In addition, the continued expansion of the depth of the reference dataset of matched morphological and genetic data for *Culicoides* specimens from India and the surrounding regions is essential to enable accurate assessments of species abundance and diversity in relation to BTV epidemiology to be made in the future and will enable the Indian *Culicoides* fauna to be placed in a global context.

## Conclusions

This study provides the foundation of the production of an updated inventory of valid species of *Culicoides* known to occur in India. The study was developed via an integrative taxonomy approach supported by publically available molecular data, in addition to new molecular data produced to strict internationally accepted metadata and quality standards [44, 112]. The study has examined the phylogenetics of *Culicoides* collected in southern India and placed results in context with both taxonomic status and the relatedness of global populations. The finding that morphological identification of potential vector species is relatively robust within this region has significant importance in interpreting previous studies of *Culicoides* carried out in southern India and in planning future studies. This taxonomic framework has the potential to be used to address a variety of areas where species-specific identification is important including relating the seasonality of adult populations to BTV outbreaks and in studies of vector capacity.

## Additional files

Additional file 1: Table S1. GenBank sequences used in genetic analyses of *Culicoides* from southern India. **Table S2.** Barcode Index Numbers (BINs) assigned within the Barcode of Life Database (BOLD) for specimens collected within this study. (DOCX 27 kb) Additional file 2: Table S3. Uncorrected percentage sequence distances, mean with range shown in parentheses. Intraspecific distances are shown in bold along the diagonal, interspecific distances are shown in the lower triangle (NA indicates comparison not possible due to singleton specimen present; number of specimens per species (n) shown in brackets with the number of specimens originating from this study; followed by the number originating from GenBank in parentheses). (XLSX 17 kb)
