# Supplementary material for: DNA barcoding and surveillance sampling strategies for Culicoides biting midges (Diptera: Ceratopogonidae) in southern India
Source: Parasit Vectors. 2016 Aug 22;9(1):461. doi: 10.1186/s13071-016-1722-z (PMC4994320; doi:10.1186/s13071-016-1722-z)
Supplement: Additional file 1: Table S1. — GenBank sequences used in genetic analyses of Culicoides from southern India. Table S2. Barcode Index Numbers (BINs) assigned within the Barcode of Life Database (BOLD) for specimens collected within this study. (DOCX 27 kb) [file 13071_2016_1722_MOESM1_ESM.docx]

**Supplementary Information 1 of 2 - Harrup et al 'DNA barcoding and surveillance sampling strategies for *Culicoides* biting midges (Diptera: Ceratopogonidae) in southern India'**

**Table S1. GenBank sequences used in genetic analyses of *Culicoides* from southern India.**

| **Species** | **Accession number** | **Reference** |
| --- | --- | --- |
| ***C. actoni* Smith, 1929** | KJ162953 | [1] |
|  | AB360971 | [2] |
| ***C. anophelis* Edwards, 1922** | KF145178 | [3] |
| ***C. asiana* Bellis, 2014*** | KJ162954-KJ162960 | [1] |
|  | AB360994-AB360995 | [2] |
| ***C. bolitinos* Meiswinkel, 1989** | AF071928-AF071931 | [4] |
|  | KJ162961-KJ162962 | [1] |
| ***C. brevitarsis* Kieffer, 1917** | KJ162966- KJ162975 | [1] |
| ***C. dubius* Arnaud, 1956** | AB361000 | [2] |
| ***C. huffi* Causey, 1938** | KF145177 | [3] |
| ***C. imicola* Kieffer, 1913** | AF069231-AF069233; AF069249 | [4] |
|  | AF078098-AF078100; AF079975-AF079979; AF080527, AF080527-AF080532; AF080534- AF080535; AF080537-AF080540; AF083044;  AJ549388-AJ549426 | [5] |
|  | AJ867223-AJ867234; EU189055-EU189057 | [6] |
|  | DQ868882-DQ868894; DQ871030 | [7] |
|  | HQ824456 | [8] |
|  | JN545055-JN545056 | [9] |
|  | KF682480-KF682481 | [10] |
|  | KJ162980-KJ162984 | [1] |
|  | AF080528, AF080529, AF080536 | Submitted, unpublished: Linton, Y.M., Mordue, A.J. and Dallas, J.F. |
| ***C. innoxius* Sen and Das Gupta, 1959** | KF145176 | [3] |
| ***C. nudipalpis* Delfinado, 1961** | KJ162989-KJ162995 | [1] |
| ***C. orientalis* Macfie, 1932** | KJ162997 | [1] |
| ***C. oxystoma* Kieffer, 1910** | AB360978-AB360985 | [2] |
|  | JN545045-JN545049; JN545052; JN545054 | [9] |
|  | KF528692-KF528694; KF682498-KF682522; KF682529-KF682533 | [10] |
| ***C. peregrinus* Kieffer, 1910** | AB361003 | [2] |
|  | KF528700 | Submitted, unpublished: He, J., Su, S., Chen, J. and Song, F. |
| ***C. subschultzei* Cornet and Brunhes, 1994** | KF682523-KF682525 | [10] |
| ****C. asiana*** (*nomen novum* for *C. asiatica* Bellis [11] (preoccupied by *C. asiaticus* Gutsevich and Smatov 1966)) specimens redescribed by Bellis et al [1] | | |

**Table S2. Barcode Index Numbers (BINs) [12] assigned within the Barcode of Life Database (BOLD) [13] for specimens collected within this study.**

| **Species** | **BIN** | **Specimen IDs** |
| --- | --- | --- |
| ***C. actoni* Smith, 1929** | BOLD:AAJ7360 | TPI:ENT:IBVNET-CULI-TN-43 |
| ***C. anophelis* Edwards, 1922** | BOLD:ACG0209 | TPI:ENT:IBVNET-CULI-TN-17;  TPI:ENT:IBVNET-CULI-TN-25;  TPI:ENT:IBVNET-CULI-TN-26;  TPI:ENT:IBVNET-CULI-TN-27;  TPI:ENT:IBVNET-CULI-TN-28;  TPI:ENT:IBVNET-CULI-TN-29;  TPI:ENT:IBVNET-CULI-TN-30;  TPI:ENT:IBVNET-CULI-TN-31;  TPI:ENT:IBVNET-CULI-TN-32 |
| ***C. brevitarsis* Kieffer, 1917** | BOLD:ACM9505 | TPI:ENT:IBVNET-CULI-TN-36;  TPI:ENT:IBVNET-CULI-TN-37;  TPI:ENT:IBVNET-CULI-TN-38;  TPI:ENT:IBVNET-CULI-TN-39;  TPI:ENT:IBVNET-CULI-TN-40;  TPI:ENT:IBVNET-CULI-TN-41;  TPI:ENT:IBVNET-CULI-TN-66;  TPI:ENT:IBVNET-CULI-TN-67;  TPI:ENT:IBVNET-CULI-TN-68 |
| ***C. huffi* Causey, 1938** | BOLD:ACM8820* | TPI:ENT:IBVNET-CULI-KA-5;  TPI:ENT:IBVNET-CULI-TN-13;  TPI:ENT:IBVNET-CULI-TN-14;  TPI:ENT:IBVNET-CULI-TN-15;  TPI:ENT:IBVNET-CULI-TN-9 |
|  | BOLD:ACG0385* | TPI:ENT:IBVNET-CULI-KA-2 |
| ***C. imicola* Kieffer, 1913** | BOLD:AAB8379 | TPI:ENT:IBVNET-CULI-KA-1;  TPI:ENT:IBVNET-CULI-TN-47;  TPI:ENT:IBVNET-CULI-TN-48;  TPI:ENT:IBVNET-CULI-TN-49;  TPI:ENT:IBVNET-CULI-TN-50;  TPI:ENT:IBVNET-CULI-TN-51 |
| ***C. innoxius* Sen and Das Gupta, 1959** | BOLD:ACG0386 | TPI:ENT:IBVNET-CULI-TN-42 |
| ***C. kepongensis*** **Lee, 1988** | BOLD:ACM9421 | TPI:ENT:IBVNET-CULI-TN-57;  TPI:ENT:IBVNET-CULI-TN-58;  TPI:ENT:IBVNET-CULI-TN-59;  TPI:ENT:IBVNET-CULI-TN-60;  TPI:ENT:IBVNET-CULI-TN-61;  TPI:ENT:IBVNET-CULI-TN-62;  TPI:ENT:IBVNET-CULI-TN-63;  TPI:ENT:IBVNET-CULI-TN-64 |
| ***C. mesghalii*** **Navai, 1973** | BOLD:ACM8766 | TPI:ENT:IBVNET-CULI-TN-18;  TPI:ENT:IBVNET-CULI-TN-19;  TPI:ENT:IBVNET-CULI-TN-20;  TPI:ENT:IBVNET-CULI-TN-21;  TPI:ENT:IBVNET-CULI-TN-22;  TPI:ENT:IBVNET-CULI-TN-23;  TPI:ENT:IBVNET-CULI-TN-24; |
| ***C. oxystoma* Kieffer, 1910** | BOLD:AAD1856 | TPI:ENT:IBVNET-CULI-TN-1;  TPI:ENT:IBVNET-CULI-TN-2;  TPI:ENT:IBVNET-CULI-TN-3;  TPI:ENT:IBVNET-CULI-TN-4;  TPI:ENT:IBVNET-CULI-TN-5;  TPI:ENT:IBVNET-CULI-TN-6;  TPI:ENT:IBVNET-CULI-TN-7;  TPI:ENT:IBVNET-CULI-TN-8 |
| ***C. similis*** **Carter, Ingram and Macfie, 1920** | BOLD:ACM9587 | TPI:ENT:IBVNET-CULI-TN-10  TPI:ENT:IBVNET-CULI-TN-11  TPI:ENT:IBVNET-CULI-TN-12  TPI:ENT:IBVNET-CULI-TN-16 |
| ***C. peliliouensis,* Tokunaga, 1936** | BOLD:ACM8856 | TPI:ENT:IBVNET-CULI-TN-33;  TPI:ENT:IBVNET-CULI-TN-34;  TPI:ENT:IBVNET-CULI-TN-35 |
| ***C. peregrinus* Kieffer, 1910** | BOLD:ACS9302 | TPI:ENT:IBVNET-CULI-TN-44;  TPI:ENT:IBVNET-CULI-TN-45;  TPI:ENT:IBVNET-CULI-TN-46;  TPI:ENT:IBVNET-CULI-TN-52;  TPI:ENT:IBVNET-CULI-TN-53;  TPI:ENT:IBVNET-CULI-TN-54;  TPI:ENT:IBVNET-CULI-TN-55;  TPI:ENT:IBVNET-CULI-TN-56 |
| **Unknown Species (I)** | BOLD:ACM8392 | TPI:ENT:IBVNET-CULI-TN-65 |
| ***** Multiple BIN numbers indicates potential new taxa: BINs are set at 3% sequence difference [12] | | |

**References**

1. Bellis G, Dyce A, Gopurenko D, Yanase T, Garros C, Labuschagne K, Mitchell A: Revision of the *Culicoides* Avaritia *Imicola* complex Khamala & Kettle (Diptera: Ceratopogonidae) from the Australasian region. *Zootaxa*. 2014; 3768(4):401.

2. Matsumoto Y, Tanase T, Tsuda T, Noda H: Species-specific mitochondrial gene rearrangements in biting midges and vector species identification. *Med Vet Entomol*. 2009; 23:47-55.

3. Archana M, Placid EDS, Jalali SK, Renukaprasad C, Rakshith O: DNA barcoding of commonly prevalent *Culicoides* midges in South India. *Indian J Anim Sci*. 2015; 85(1):37-39.

4. Linton YM, Mordue AJ, Cruickshank RH, Meiswinkel R, Mellor PS, Dallas JF: Phylogenetic analysis of the mitochondrial cytochrome oxidase subunit I gene of five species of the *Culicoides imicola* species complex. *Med Vet Entomol*. 2002; 16:139-146.

5. Dallas JF, Cruickshank RH, Linton YM, Nolan DV, Patakakis M, Braverman Y, Capela M, Capela R, Pena I, Meiswinkel R *et al*: Phylogenetic status and matrilineal structure of the biting midge, *Culicoides imicol*a, in Portugal, Rhodes and Israel. *Med Vet EntomolI.* 2003; 17:379-387.

6. Nolan DV, Dallas JF, Piertney SB, Mordue (Luntz) AJ: Inclusion and range expansion in the bluetongue vector *Culicoides imicola* in the Mediterranean basin: a phylogeographic analysis. *Med Vet Entomol*. 2008; 22:340-351.

7. Calvo JH, Calvete C, Martinez-Royo A, Estrada R, Miranda MA, Borràs D, Sarto IMV, Pages N, Delgado JA, Collantes F *et al*: Variations in the mitochondrial cytochrome c oxidase subunit I gene indicate northward expanding populations of *Culicoides imicola* in Spain. *Bull Entomol Res*. 2009; 99(6):583-591.

8. Wenk CE, Kaufmann C, Schaffner F, Mathis A: Molecular characterization of Swiss Ceratopogonidae (Diptera) and evaluation of real-time PCR assays for the identification of *Culicoides* biting midges. *Vet Parasitol*. 2012; 184(2-4):258-266.

9. Morag N, Saroya Y, Braverman Y, Klement E, Gottlieb Y: Molecular identification, phylogenetic status and geographic distribution of *Culicoides oxystoma* (Diptera: Ceratopogonidae) in Israel. *PLoS ONE*. 2012; 7:e33610.

10. Bakoum MT, Fall M, Fall AG, Bellis GA, Gottlieb Y, Labuschagne K, Venter GJ, Diop M, Mall I, Seck MT *et al*: First record of *Culicoides oxystoma* Kieffer and diversity of species within the Schultzei Group of *Culicoides* Latreille (Diptera: Ceratopogonidae) biting midges in Senegal. *PLoS ONE.* 2013; 8(12):e84316.

11. Bellis GA, Halling L, Anderson SJ: Pictorial key to adult female *Culicoides* Latreille, 1809 (Diptera: Ceratopogonidae) from the Northern Territory, Western Australia and South Australia. *Austral Ent*. 2014; 54(1):28-59.

12. Ratnasingham S, Hebert PDN: A DNA-Based Registry for All Animal Species: The Barcode Index Number (BIN) System. *PLoS ONE* 2013, 8(7):e66213.

13. Ratnasingham S, Hebert PDN: BOLD: The Barcode of Life Data System (www.barcodinglife.org). Mol Ecol Notes 2007, 7:355-364.
